# A Color Restoration Algorithm for Diffractive Optical Images of Membrane Camera

**DOI:** 10.3390/s21041053

**Published:** 2021-02-04

**Authors:** Yanlei Du, Xiaofeng Yang, Yiping Ma, Chunxue Xu

**Affiliations:** 1 State Key Laboratory of Remote Sensing Science, Aerospace Information Research Institute, Chinese Academy of Sciences, Beijing 100101, China; duyl01@radi.ac.cn; 2 Department of Electronic Engineering, Tsinghua University, Beijing 100084, China; 3 Key Laboratory of Earth Observation of Hainan Province, Sanya 572029, China; 4 Beijing Municipal Commission of Planning and Natural Resources, Beijing 101160, China; 5 College of Earth, Ocean, and Atmospheric Sciences, Oregon State University, Corvallis, OR 97331, USA; xuch@oregonstate.edu

**Keywords:** color restoration, diffractive optical images, membrane camera

## Abstract

In order to verify the technology of the membrane diffractive imaging system for Chinese next generation geo-stationary earth orbit (GEO) satellite, a series of ground experiments have been carried out using a membrane optical camera with 80 mm aperture (Φ80) lens. The inherent chromatic aberration due to diffractive imaging appears in the obtained data. To address the issue, an effective color restoration algorithm framework by matching, tailoring, and non-linearly stretching the image histograms is proposed in this letter. Experimental results show the proposed approach has good performances in color restoration of the diffractive optical images than previous methods. The effectiveness and robustness of the algorithm are also quantitatively assessed using various color deviation indexes. The results indicate that the chromatic aberration of diffractive images can be effectively removed by about 85%. Also, the proposed method presents reasonable computational efficiency.

## 1. Introduction

The geo-stationary earth orbit (GEO) satellite can provide remote sensing image data with wide observation range and high temporal resolution [1,2]. However, because of the high orbital altitude, achieving high spatial resolution is still a challenge for the GEO optical system. Theoretically, to achieve the meter-level resolution at the GEO height, the aperture of camera can be up to 20 m [3,4]. Considering the support and control systems, the size and weight of satellite would be extremely large. This is prohibitive for the engineering fabrication and deployment of traditional optical camera.

To alleviate the technique contradictions of GEO remote sensing system, a novel membrane optical system has been proposed and seen increasing interests in relevant studies [5,6,7,8,9]. Unlike the traditional reflective optics, the membrane camera adopts the transmissive diffractive imaging mechanism. The diffractive primary is manufactured by the macromolecule polymer material which has the merits of light weight, low cost, and high flexibility. Thus, large aperture but light weight of the optical imaging system can be achieved. Also, easy deployment (light and packable) of the membrane optical system virtually eliminates the tight surface shape tolerances and significantly reduces the complexity of the control architecture faced by conventional large reflecting apertures [9,10]. Several ongoing missions equipped with the diffractive membrane elements include the “Eyeglass” telescope mission, the Membrane Optical Imager Real-time Exploitation (MOIRE) mission and the FalconSat-7 mission [9,11,12,13]. However, the inherent spectral dispersion and wavefront distortion of diffractive primary lead to prominent degradations of the diffractive images, e.g., image blurring, image hazing, and color distortion [14,15]. In addition, considering the long optical distance of GEO observation, the wavelength correlation of atmospheric scattering would also cause image chromatic aberration.

Chinese next generation GEO remote sensing satellites plan to equip the diffractive membrane optical system for earth observation with ultra-high spatial resolution. Ground experiments have been carried out for technique verification using a membrane camera with an 80 mm aperture (Φ80) primary. Image degradations mentioned above also appear in the image data. Particularly, without the chromatic aberration correction (CAC) system, the color distortions of data are severe. For the development of real-time processing CAC system and the follow-up applications of remote sensing analyses, an effective and efficient color restoration algorithm is in the imperative demand.

Previous studies of image restoration for diffractive imaging mainly focused on improving image definition using single-band data [14,16,17]. Image color restoration, or white balance, has been extensively studied and applied to the conventional reflection optical images [18]. Classic and efficient color restoration algorithms include the gray world algorithm (GWA), the perfect reflection method (PRM), and the white patch Retinex (WPR). [19,20]. These methods attempt to achieve human visual color constancy on the basis of various assumptions. The GWA assumes that the average reflectance in a scene for each channel under a neutral light source should be achromatic [21]. The PRM utilizes the maximum value of each image channel to compensate the color cast [22]. The WPR is based on the Retinex theory, which argues that perceived white is associated with the maximum cone signals [19,23,24]. However, these classic approaches may not satisfactorily work for certain conditions [25]. Thus, several state-of-art white balance methods were developed over the last two decades [25,26,27,28,29,30,31]. Huo et al. proposed a robust automatic white balance (RAWB) algorithm based on the color temperature estimation using extracting gray color points [25]. Limare et al. put forward a simplest color balance (SCB) method by stretching the digital number (DN) values of all channels as much as possible [26]. Recently, Mahmoud Afifi and Michael S. Brown published two novel method of image white balance, i.e., the interactive white balancing (IWB) method [29,31] and the deep white balance editing (DWBE) method, in CIC-2020 and CVPR-2020, respectively [30]. The IWB is on a basis of the nonlinear color-mapping functions [31] and enables an interactive white balance manipulation of user by selecting colors from images [29]. The implementation of DWBE is based on a novel deep learning framework. The above mentioned methods of image color restoration were carefully tried and assessed for the diffractive images with chromatic aberration.

In this letter, we propose an effective and tractable color restoration algorithm for diffractive optical images of membrane camera. According to the definitions of color deviation indexes, the statistic information among band histograms and spectral characteristics of the diffractive images are exploited in the algorithm. It incorporates operations of histogram matching, tailoring, and stretching. Thus we name it the HMTS method. Performances of the approach in color restoration are evaluated qualitatively and quantitatively. Experiments indicate that the proposed method shows tractability and robustness in chromatic aberration correction of diffractive images. This paper provides a technical support for the deployment of future diffractive membrane camera with large aperture at the geostationary orbit and the applications of earth observation.

## 2. Ground Experiments and Diffractive Image Data

**Figure 1:**
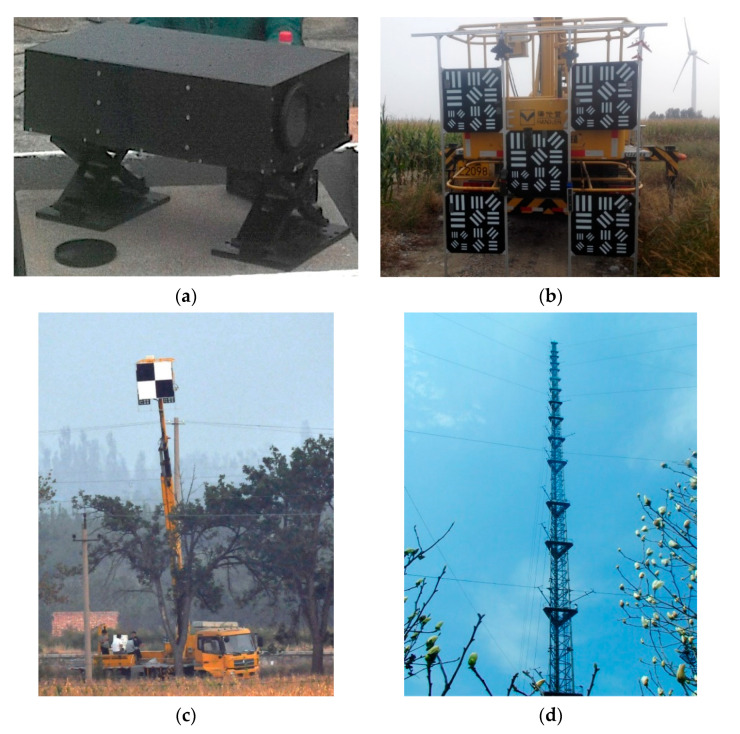
(**a**) Φ80 mm diffractive membrane camera. (**b**) Bar target loaded on the overhead working truck. (**c**) Work scene of using the overhead working truck and (**d**) the meteorological tower for loading the diffractive membrane camera (DMC) during the ground experiments.

In order to validate the imaging capability of diffractive membrane camera, several ground experiments have been accomplished from September 2017 to October 2019 under the sponsorship of Chinese ministry of science and technology (CMST). A diffractive membrane camera (DMC) with Φ80 mm primary was fabricated and used in experiments, which is shown in Figure 1a. The focal length of the DMC is 639.7 mm. It can achieve color imaging within the spectral range from 0.486 μm to 0.65 μm and at the distances from 100 m to infinity. In lab measurements, the DMC has diffraction efficiency of about 25%. Before the ground experiments, the sensor of the DMC was strictly calibrated in the laboratory to make sure the systematic biases are completely eliminated. The DMC and imaging targets were loaded on a folding-jib overhead working truck or a meteorological observation tower for imaging at various distances and imaging conditions. Figure 1b shows the bar target loaded on the overhead working truck. Figure 1c shows the work scene of using the overhead working truck during the ground experiments at the Huailai remote sensing comprehensive experimental site in Hebei province of China. Figure 1d presents the meteorological tower of the Institute of Atmospheric Physics, Chinese Academy of Sciences, used in the third ground experiment for loading the DMC. Synchronously, a traditional lens with the focal length of 647 mm and the Φ80 mm aperture was also employed for reference imaging. The reference camera used the same back-end image processing system as the DMC. Particularly, in the third ground experiments, to simulate the imaging condition on the GEO orbit, the DMC was loaded on the meteorological tower, which is more than 300 m high. The targets were placed on the ground. The meteorological tower can only support one researcher to work onboard. Considering the weight of camera systems and manual operations at high elevation, it was not safe to frequently switch operations between the DMC and the reference camera. As a result, very few reference images were synchronously obtained in the third ground experiments.

**Figure 2:**
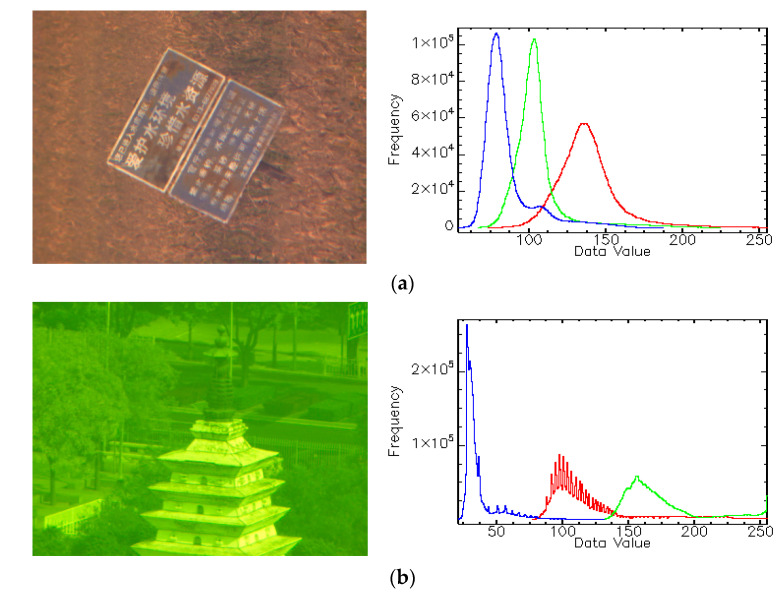
Color distorted images of the DMC and the corresponding histograms. (**a**) Test image 1. (**b**) Test image 2.

During the ground experiments, more than 20,000 diffractive images were obtained with various imaging parameter settings. Basically, the obtained diffractive images can be divided into two categories: The images of artificial targets (including various bar and resolution targets, toy models, and printed images) and the images of natural scenes (random scenes around the experimental site). Based on these image data, the imaging quality of the DMC was assessed using the spatial resolution, the modulation transfer function (MTF), and the signal to noise ratio (SNR), etc. Numerical results indicate that the image degradations appear in the single-band DMC images, which are expected and similar to the MOIRE mission [9]. Previous studies have suggested various empirical and physical-based restoration methods for diffractive images [14,15]. After implementing certain restoration approaches to the degraded single-band images, we found the qualities of DMC images basically meet the mission requirements in terms of the spatial resolution, the MTF and the SNR. However, due to the missing of chromatic aberration correction system of the DMC, serious color distortions are observed in the color diffractive images. As interpreted previously, this is resulted from the spectral distortion of diffractive primary and the wavelength correlation of atmospheric scattering. The chromatic aberration can be up to larger than 60%. Particularly, in the third ground experiment, because of the misuse of optical filter, all produced images present greenish color. Figure 2 shows two color distorted images obtained in ground experiments and the corresponding histograms. It is seen that the RGB histograms remarkably disperse in the grayscale. Notably, in the third ground experiment, because of the misuse of optical filter, all produced images present greenish color. Therefore, such color deviations can be seen as the superimposition of artificial distortion and inherent chromatic aberration of diffractive imaging. The greenish distortion has dependency of imaging circumstance which cannot be systematically removed. In addition, the atmospheric scattering and relatively low diffraction efficiency also cause the hazing effect of images so that the image visual quality would be diminished. Therefore, it is significant to perform color restoration to the diffractive images before remote sensing applications.

## 3. Methodology

In this section, we are presenting a color restoration algorithm for the diffractive images with color distortion. The aim of the color restoration is to make the images in conformity with the human visual system, so as to improve their interpretation value. Here we firstly introduce several color deviation indexes as the theoretical basis and evaluation criteria of the image color restoration.

### 3.1. Color Deviation Indexes

#### 3.1.1. Mean Dispersion Index

The classic gray world assumption argues that the averages of digital number (DN) values over the entire image of R, G, and B channels are nearly equivalent. Thus, based on the GWA hypothesis, we propose a mean dispersion index (MDI) to evaluate the image color deviation. For an image with no color deviation, the GWA considers the differences among the mean values of DN for various channels are much smaller than the mean value of all DNs of the image [19]. Thus, we define the MDI as

(1) $$\mathrm{MDI}=\left|\frac{\langle I_{R}\rangle }{\langle I_{S}\rangle }-\frac{1}{3}\right|+\left|\frac{\langle I_{G}\rangle }{\langle I_{S}\rangle }-\frac{1}{3}\right|+\left|\frac{\langle I_{B}\rangle }{\langle I_{S}\rangle }-\frac{1}{3}\right|$$

where *I* represents the DN value at each pixel. $\langle I_{R}\rangle$, $\langle I_{G}\rangle$ and $\langle I_{B}\rangle$ represent the mean DN values of each channel in the RGB color space. $\langle I_{S}\rangle$ represents the summation of the mean DN values of all three channels. They are defined as

(2) $$\langle I_{k}\rangle =\frac{1}{M}\sum_{i=1}^{M}I_{k}\left[i\right]\begin{array}{cc} & k=R,G,B \end{array}$$

(3) $$\langle I_{S}\rangle =\langle I_{R}\rangle +\langle I_{G}\rangle +\langle I_{B}\rangle$$

where *M* denotes the number of image pixels. From the definition, one can see the MDI is normalized between 0 and 1. For a color image, the closer the MDI is to 0, the less color deviation the image has.

#### 3.1.2. Histogram Overlap Area

Histogram overlap area (HOA) is a commonly used index for evaluating the color deviation of image [32]. It describes the consistency of three channels in RGB color space and has the definition as

(4) $$\mathrm{HOA}=\sum_{i=0}^{255}\min\left(h_{R}\left[i\right],h_{G}\left[i\right],h_{B}\left[i\right]\right)$$

with

(5) $$h_{k}\left[i\right]=\frac{H_{k}\left[i\right]}{M}\begin{array}{cc} & k=R,G,B \end{array}$$

(6) $$H_{k}\left[i\right]=number\left(I_{k}=i\right)\begin{array}{cc} & k=R,G,B \end{array}$$

where $h_{k}$ and $H_{k}$ represent the probability density histogram and histogram of the corresponding channel, respectively. number() represents the statistic function for counting times. Unlike the MDI, an image with the HOA close to 1 has less color deviation.

#### 3.1.3. CIEDE2000 Chromatic Aberration Coefficient

The CIEDE2000 is the newest standard chromatic aberration coefficient proposed by the international commission on illumination (CIE). It can quantitatively evaluate the difference between two colors with high accuracy. Moreover, the evaluation result is closer to the human visual system than previous coefficients. Thus, we employ the CIEDE2000 to assess the color restoration effect of the proposed algorithm. The computation of the CIEDE2000 is summarized in [33].

### 3.2. The HMTS Algorithm

To address the chromatic aberration issue of the DMC images as shown in Figure 2, we propose a color restoration algorithm framework in this letter. It incorporates the operations of matching, tailoring, and non-linearly stretching the image histograms. Thus, we name it the HMTS algorithm. The main idea of this algorithm is to exploit the spectral and statistic information of diffractive images to achieve large HOA and small MDI of the restored images. Specifically, the histogram matching (HM) is to improve the channel consistency that complies with the gray world assumption and human visual sense. Tailoring and stretching (TS) of channel histograms can suppress the noises induced by the HM and improve the image contrast. The implementing steps of the HMTS algorithm are listed as follows.

**Figure 3:**
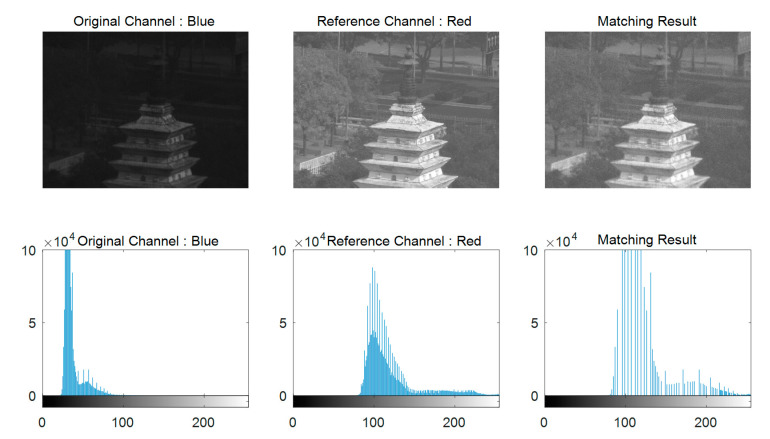
Histogram matching between channels of the diffractive image with chromatic aberration.

1. Input the diffractive image with color deviation; and
2. Compute the mean DN values of each channel (i.e., $\langle I_{R}\rangle$, $\langle I_{G}\rangle$, and $\langle I_{B}\rangle$) using (2).
3. Perform histogram matching, with selecting the channel with medium average as a reference, to the other two channels. Histogram matching is a commonly used method for image enhancement by matching the image histogram to a reference image. In this work, the inter-channel HM can significantly eliminate the luminance deviations among channels of diffractive images. Since the HM is a well-developed algorithm of image processing, one can refer to [18] for the details of HM. For the sake of simplicity, here we show the results of inter-channel HM for one of the test images shown in Figure 3. From the figure, one can see that the brightness of the original blue channel is significantly increased after the HM to the red channel. The histogram of blue channel is reformulated as that of the red one. However, as shown in Figure 3, it is also noted that the image noise of blue channel is also enlarged due to the HM operation. This phenomenon was also reported in [18]. These noises generally have very large or very small DN values. In other words, they concentrate at two ends of the grayscale histogram. A typical way to remove the image noises is to perform filtering. However, image filtering would be time consuming, particularly for the remote sensing images. Considering our motivation of supporting the real-time chromatic aberration correction system for the membrane diffractive camera, we utilize the histogram tailoring at two histogram ends to efficiently suppress the noise. Then, for the tailored histogram, we implement a non-linear histogram stretching procedure to improve the image contrast induced by the hazing effect of diffractive imaging. The details of histogram tailoring and stretching are illustrated in the following step.
4. Tailor and non-linearly stretch the histogram of each channel using
  (7) $$g_{k}\left(x,y\right)=\{\begin{array}{ccc} 0, & 0<f_{k}\left(x,y\right)<I_{k,bm} & \\ Histogram\;Stretching, & I_{k,bm}\leq f_{k}\left(x,y\right)\leq I_{k,tp} & k=R,G,B\\ 255, & I_{k,tp}<f_{k}\left(x,y\right)<255 & \end{array}$$
  where $f_{k}\left(x,y\right)$ and $g_{k}\left(x,y\right)$ denote the DN of pixel at (x,y) of original and restored images for the corresponding channel. $I_{k,bm}$ and $I_{k,tp}$ are the bottom and top DN cutoffs which are generally determined by the cutting percentage between 0.01% and 2%. Here the cutting percentage is defined as the number of pixels with DN smaller than $I_{k,bm}$ and larger than $I_{k,tp}$ divided by the total pixel numbers. Thus, the DN cutoffs can be given by the gray cumulative histogram for a specific cutting percentage. For the non-linear histogram stretching of the tailored histogram between DN cutoffs, we follow [34] and use the quadratic transformation function to stretch the histogram, which is described as follows
  (8) $$I_{k,str.}\left(I_{k}\right)=\alpha I_{k}{}^{2}+\beta I_{k}+\gamma \begin{array}{cc} & k=R,G,B \end{array}$$
  where $I_{k,str.}$ denotes the new DN value after gray transformation. *α*, *β* and *γ* are the coefficients which can be computed following
  (9) $$\{\begin{array}{c} I_{k,str.}\left(I_{k,bm}\right)=\alpha I_{k,bm}^{2}+\beta I_{k,bm}+\gamma =0\\ I_{k,str.}\left(I_{k,mn}\right)=\alpha I_{k,mn}^{2}+\beta I_{k,mn}+\gamma =\eta \frac{255}{2}+\left(1-\eta \right)I_{k,mn}\\ I_{k,str.}\left(I_{k,tp}\right)=\alpha I_{k,tp}^{2}+\beta I_{k,tp}+\gamma =255 \end{array}$$
  (10) [αβγ]=[Ik,bm2Ik,bm1Ik,mn2Ik,mn1Ik,tp2Ik,tp1]−1[0η⋅2552+(1−η)Ik,mn255]
  where $I_{k,mn}$ is the mean DN value of corresponding channel. η∈[0,1] is the brightness weight which is selected at 0.4 in this study.
  There are two reasons to account for the process of histogram tailoring and stretching. Firstly, in the previous step of histogram matching, the image noise of the matching channel could be enlarged by matching to the reference channel. Those noise signals typically have extreme values. Thus, histogram tailoring at the two ends of grayscale histogram would be conducive to suppress the noise. Secondly, implementing the histogram tailoring also aims to improve the image contrast and definition to remove the hazing effect. From (7), it is noted that both histogram tailoring and stretching depend on the bottom and top DN cutoffs of histogram, i.e., $I_{k,bm}$ and $I_{k,tp}$. The selection of the DN cutoffs determines the final color restoration effects. Theoretically, removing the hazing effect would increase the image depth of filed so that the image information would be increased. However, on the other hand, too much histogram tailoring would reduce the image information. Thus, the basic principle of selecting the histogram cutoffs is to reduce image noise and enhance the contrast to largest extent with losing less image information. As a result, we repeatedly perform histogram tailoring and non-linear stretching with various cutoffs to obtain the image with maximum information. The initial cutting percentage is given as 0.01%. Particularly, the information entropy (IE) is employed to evaluate the image information and be a stopping criterion. The IE is defined as [35]
  (11) $$E=-\sum_{i=0}^{255}p_{i}\cdot \log_{2}p_{i}$$
  where $p_{i}$ denotes the probability of grayscale *i*, which can be calculated from the image histogram (see (5) and (6)) as follows
  (12) $$p_{i}=\frac{\sum_{k}number\left(I_{k}=i\right)}{3\times M}\begin{array}{cc} & k=R,G,B \end{array}$$
  By repeatedly performing this procedure of histogram tailoring and non-linear stretching using various cutting percentages in a grid one by one, the image with largest IE would be obtained. The corresponding cutting percentage can be used to remove noises but retain the useful information. The detailed algorithm of histogram tailoring and stretching is described as follows (Algorithm 1).
**Algorithm 1**. Histogram tailoring and stretching.**Inputs:** diffractive image after inter-channel HM,     initial cutting percentage cp = 0.1%,     initial information entropy ie = 0; **Outputs:** color restored image.1: **for** iteration *i*
**do**2:  **if**
*cp* ≤ 2% & *ie*(*i*) ≥ *ie*(*i*-1) **do**3:   compute gray cumulative histograms (GCH) and $I_{k,mn}$ of each channel;4:   compute $I_{k,bm}$ and $I_{k,tp}$ using *cp* and GCH for each channel;5:   compute *α*, *β* and *γ* using (10);6:   do gray transformation using (9);7:   compute image information entropy *ie*;8:   *cp* ← *cp*+0.1%;9:  **else**10:   **break**;11:  **end if**12: **end for**13: **return** color restored image
5. Output the restored image.

## 4. Results and Evaluations

**Figure 4:**
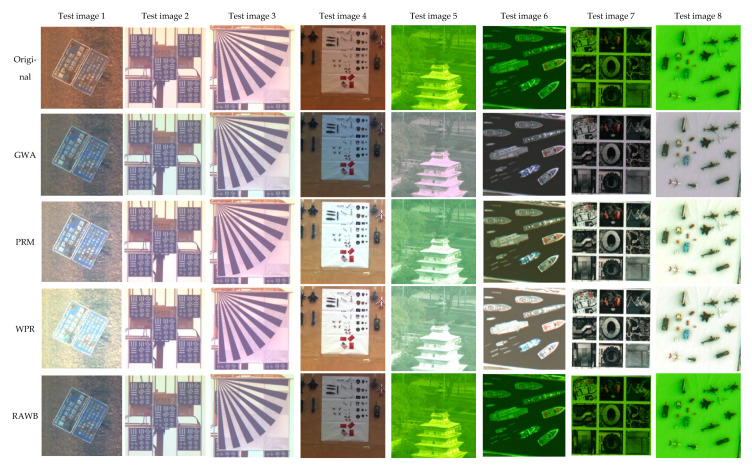

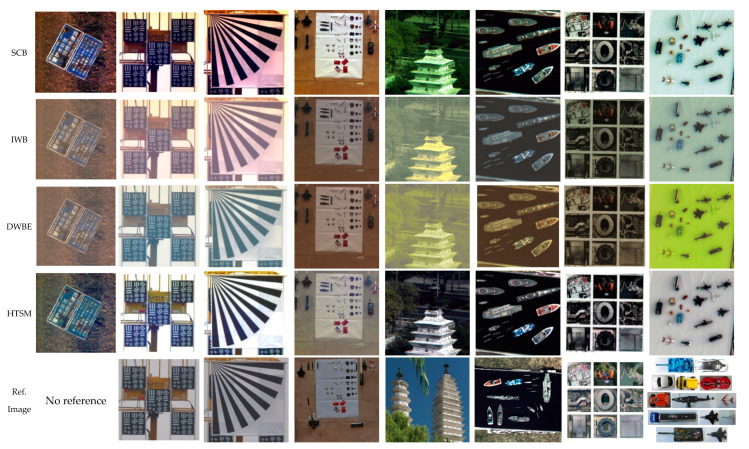
Comparison of color restoration effect of various algorithms for diffractive images.

In Figure 4, the proposed HMTS algorithm is implemented to the color distorted DMC images. The results are also compared to those using the GWA, the PRM, the WPR, the RAWB, the SCB, the IWB, and the DWBE. In particular, the auto white-balance correction module in the IWB was practically utilized. The DWBE was trained on the dataset which is available on http://cvil.eecs.yorku.ca/projects/public_html/sRGB_WB_correction/dataset.html (accessed on 1 February 2021). To comprehensively assess the performances of various methods in restoring the chromatic aberration of diffractive images, total 8 test diffractive images are used which involve all three ground experiments. For a fair and objective evaluation, we also present the corresponding reference images. However, for the test image 1 (warning board in the field) obtained in the first experiment, the reference camera unfortunately failed to follow up and capture image. In addition, as introduced in Section 2, due to the limitation of experimental environment, very few reference images were synchronously obtained in the third ground experiments. Thus, for those diffractive images with no reference images obtained, we used other images to show the original color of targets. Notably, the reference image is the digital image for printing, in which the colors are slightly different from those in the printed target for imaging. Table 1 presents the MDI and HOA indexes of original images and color restored images using various algorithms, which are presented in blue and red, respectively. Particularly, for the convenience of evaluation and discussion, the smallest two MDIs and the largest two HOAs are emphasized in black bold in Table 1.

From Figure 4, one could see that the proposed HMTS method in general has the best color restoration performance for the test diffractive images. The image visual effects are close to the reference images. This is also illustrated in Table 1 that the results of HMTS basically have smallest MDIs and largest HOAs. In addition, with considering the hazing effect due to low diffraction efficiency and atmospheric scattering, the restored images of HMTS present large image contrast and depth of field. For the test images 2 and 3, considering the cloudy and haze weather of the imaging circumstance (note this in the reference images), the diffractive images after color restoration are slightly overexposed due to the sky background. For the comparison methods, the performances of the GWA method are barely satisfactory. The chromatic aberrations in test images 1, 6, 7, and 8 are mostly removed by the GWA, which are also indicated by the color deviation indexes shown in Table 1. Notably, since the definition of MDI index obeys the basic assumption of the GWA, all the restored images using the GWA have very small MDI. Based on the visual evaluation and the quantitative indexes, the PRM and WPR also have relatively good performances for test images 1 and 7 and test images 4, 7, and 8, respectively. The RAWB method yields acceptable results for test images 1 and 4. However, it almost gives no improvements to the diffractive images with greenish color deviation obtained in the third ground experiment. For the SCB method, since it stretches the histograms of all channels as much as it can be, all the restored images have very large contrasts. In terms of the MDI, the SCB algorithm has relatively good performance for test images 6, 7 and 8. The IWB and DWBE also have different performances on various test images. It is seen that the IWB has better color restoration effects on test image 6 and 8, while the DWBE scores on test image 2 and 3. In addition, for the test image 5 (the white tower), it should be note that almost all comparison methods fail to give satisfying results. According to the HOA index, the restored result of test image 5 using the HMTS has much higher HOA than those of other comparison approaches.

In Table 2, we present the CPU time consumption of various algorithms to compare their computational efficiencies. The color restoration experiments are carried out on the device equipped with the Intel Core i7-8700 CPU at 3.20 GHz and 32 GB of memory capacity. According to the average time consumptions, one could see that the WPR is the fastest algorithm among the methods, followed closely by the PRM and our proposed HMTS. The DWBE and the RAWB have lowest computational efficiency. Considering the motivations of good color restoration performance and real-time processing capability for the CAC system development, the proposed HMTS algorithm has reasonable computational efficiency.

**Figure 5:**
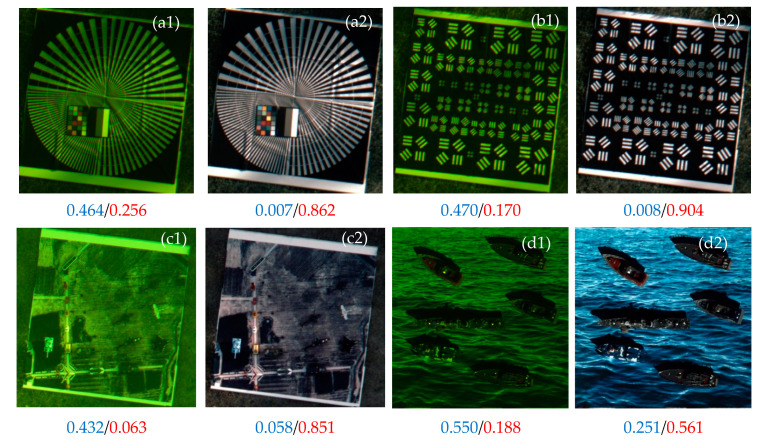
Results of the proposed HMTS for diffractive images with chromatic aberration in the third ground experiment. Blue and red numbers denote the MDI and HOA of the corresponding images.

To further verify the robustness of the HMTS algorithm, more experiments are conducted on the diffractive images obtained from the third ground experiments with serious color deviation. In Figure 5, the HMTS algorithm is implemented to various resolution targets and image targets of ocean scenes and land scenes. The corresponding MDI and HOA of images are computed as well. By comparing the image before and after processing, one can see that the color of the diffractive images are well restored and the image visual effects are significantly improved. It is also noted that the HOAs increase and the MDIs decrease for all test images after implementing the HMTS algorithm. Considering all experimental images shown in Figure 4 and Figure 5 and taking the statistical averages of the color deviation indexes, it is found that the average MDI decreases from 0.349 to 0.037, and the average HOA increases from 0.154 to 0.776, after implementing the color restoration with HMTS. These indicate that the HMTS algorithm has good performance and strong robustness of color restoration for diffractive images of various scenes and targets.

Moreover, we utilize a standard color-checker (SCC) chart produced by the X-Rite to further assess the effects of the HMTS in color restoration. The CIEDE2000 coefficients are computed referring to the SSC image taken by the reference camera. Moreover, several comparison methods, e.g., the GWA, the WPR, the SCB and the IWB, which have relatively good color restoration performances for the images obtained in the third ground experiment, are also taken into comparisons. In Table 3, comparisons of the SCC images and the corresponding CIEDE2000 coefficients before and after implementing color restorations are presented. As shown in the table, comparing to the other four methods, the SCC images after implementing the HMTS have closer visual effects to those taken by reference camera. The mean value of the CIEDE 2000 coefficients of SCC images after color restoration using the HMTS is only about one seventh of that before color restoration, which is the smallest one among the comparison methods. This indicates that about 85% of the color deviations in diffractive images have been removed.

## 5. Conclusions

This letter presents a color restoration algorithm, the HMTS, for the diffractive optical images of membrane camera, which is planned to be equipped on Chinese next generation GEO remote sensing satellites. The HMTS algorithm incorporates steps of matching, tailoring and non-linearly stretching image histograms to achieve color restoration. Experimental results show that the restored images using the proposed algorithm comply with human visual sense well. The color deviation indexes also quantitatively validate the good performance and robustness of the HMTS in restoring the chromatic aberration of diffractive images. In addition, the proposed method has reasonable computational efficiency. Note that the HMTS method has the theoretical basis of channel consistency, which indicates it is a global automatic white balance (AWB) method. Thus, it would be invalid for the images with homogenous scenes which have quite different spectral properties at different channels. In fact, as stated in [25], this can be seen as an intrinsic limit of the global AWB methods for the color distorted images dominated by only one or two colors. Yet, this situation is less likely to happen for the earth observation of GEO satellite, because the wide imaging range would include diverse targets and scenes. Currently, with the development of the CAC system, this study would provide the technical support for the deployment of future GEO diffractive optical system and the relevant remote sensing applications.

## Figures and Tables

**Table 1:** Comparison of the mean dispersion index (MDI) and histogram overlap area (HOA) of color restoration images using various algorithms. Blue and red numbers denote the MDIs and HOAs of the corresponding results with the smallest two MDIs and the largest two HOAs being emphasized in black bold.

	Test Image 1	Test Image 2	Test Image 3	Test Image 4	Test Image 5	Test Image 6	Test Image 7	Test Image 8
	(MDI/HOA)							
Original image	0.172/0.132	0.055/0.366	0.044/0.299	0.217/0.166	0.450/3.976× 10^−6^	0.449/0.012	0.473/0.186	0.411/0.012
GWA	**2.887 × 10^−4^**/0.699	**1.887 × 10^−4^**/0.415	**0.002**/0.431	**6.948 × 10^−4^**/0.161	**0.003/0.594**	**2.803 × 10^−4^** **/0.549**	**4.980 × 10^−4^** **/0.864**	**5.080 × 10^−4^**/0.556
PRM	0.016/0.607	0.025/0.439	0.033/0.380	0.067/0.230	0.128/0.171	0.121/0.237	0.035/0.831	0.044/0.403
WPR	0.045/0.456	0.022/**0.484**	0.037/0.433	0.090/**0.389**	0.070/0.335	0.047/0.302	0.022/0.837	0.029/**0.619**
RAWB	0.017/**0.779**	0.005/0.348	0.005/0.369	0.088/0.372	0.450/3.975 × 10^−6^	0.450/0.012	0.474/0.185	0.411/0.012
SCB	0.159/0.106	0.059/0.139	0.084/0.122	0.129/0.083	0.205/0.050	0.027/0.105	0.031/0.055	0.036/0.075
IWB	0.107/0.236	0.061/0.406	0.052/0.298	0.110/0.269	0.103/0.280	0.010/0.416	0.083/0.680	0.047/0.353
DWBE	0.088/0.288	0.013/0.448	0.011/**0.518**	0.145/0.154	0.167/0.087	0.188/0.112	0.110/0.670	0.330/0.171
HMTS	**0.005/0.923**	**9.301 × 10^−4^/0.769**	**0.002/0.781**	**0.043/** **0** **.385**	**0.029/0.847**	**0.037/0.663**	**0.006/0.904**	**0.001/0.862**
Reference image	/	0.015/0.682	0.008/0.634	0.066/0.193	/	0.017/0.432	0.004/0.887	/

**Table 2:** Comparison of the CPU time consumptions of various algorithms in color restorations for the experimental diffractive images.

	Test Image 1	Test Image 2	Test Image 3	Test Image 4	Test Image 5	Test Image 6	Test Image 7	Test Image 8	Average
GWA	0.89	0.61	0.67	0.80	0.83	0.70	0.67	0.70	**0.73**
PRM	0.27	0.17	0.14	0.55	0.33	0.19	0.16	0.38	**0.27**
WPR	0.08	0.01	0.02	0.08	0.09	0.02	0.02	0.03	**0.04**
RAWB	7.34	3.68	4.52	5.05	4.20	2.52	2.38	2.26	**3.99**
SCB	2.11	0.55	0.56	1.53	1.98	1.03	0.98	0.80	**1.19**
IWB	1.75	0.83	0.80	1.03	1.20	0.91	0.90	1.28	**1.08**
DWBE	7.20	1.60	1.56	4.01	4.96	3.18	3.08	5.05	**3.83**
HMTS	0.69	0.04	0.14	0.59	0.67	0.33	0.22	0.23	**0.36**

**Table 3:** Comparisons of the colorimetric chart images and the corresponding CIEDE2000 coefficients before and after implementing color restoration using various methods.

	SCC Images (Reference Camera)	Before Color Restoration		After Color Restoration									
		SCC Images	CIEDE 2000	GWA		WPR		SCB		IWB		HMTS	
				SCC Images	CIEDE 2000	SCC Images	CIEDE 2000	SCC Images	CIEDE 2000	SCC Images	CIEDE 2000	SCC Images	CIEDE 2000
1	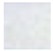	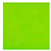	26.4894	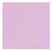	9.7787	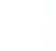	2.4682	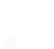	2.4583	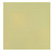	13.6672	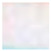	1.3466
2	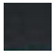	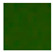	26.9623	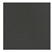	9.0362	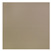	26.6412	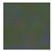	16.3484	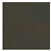	11.4082	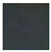	6.8833
3	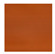	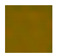	18.6755	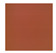	7.5564	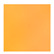	18.0491	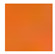	8.1791	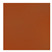	2.4199	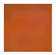	2.7182
4	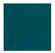	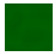	31.0801	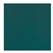	6.3244	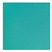	18.6522	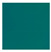	8.0410	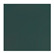	10.7433	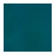	1.5030
5	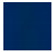	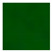	35.8459	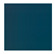	12.3313	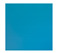	26.3896	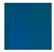	13.7142	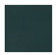	17.8162	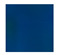	6.2752
6	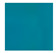	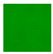	33.9223	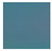	9.0784	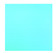	17.9978	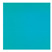	7.6340	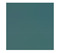	9.0625	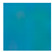	3.5805
7	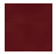	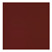	28.3517	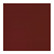	6.5519	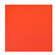	25.3978	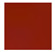	13.9728	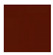	14.0102	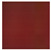	7.9623
Mean Value	/	/	28.7610	/	8.6653	/	19.3708	/	10.0497	/	11.3039	/	4.3242

## Data Availability

The data presented in this study are available on request from the corresponding authors. The data are not publicly available due to the funding policy.
